# A dual telescoping lag screw nailing system for intertrochanteric fractures: retrospective analysis of clinical and radiologic outcomes

**DOI:** 10.1007/s00590-024-03906-w

**Published:** 2024-03-31

**Authors:** Matteo Innocenti, Filippo Leggieri, Gregorio Secci, Christian Carulli, Armando Del Prete, Roberto Civinini

**Affiliations:** Department of General Orthopedic, University of Florence, A.O.U. Careggi CTO - Largo Palagi 1, 50139 Florence, Italy

**Keywords:** Hip fractures, Intramedullary nailing, Telescoping screw, Lateral protrusion, Cut-out

## Abstract

**Background and purpose:**

The aim of this study was to evaluate whether this system is associated with a reduced rate of failure and complications in patients treated for proximal femoral fractures with intramedullary nailing.

**Materials and methods:**

742 Patients with AO-OTA 31-A intertrochanteric fractures were enrolled at a single Institution. Functional evaluation was assessed through the Functional Independence Measure (FIM™) instrument and Parker’s New Mobility Score (NMS). Radiological follow-up included the degree of the reduction according to the Baumgartner criteria, the Tip-Apex Distance, and the shortening of the telescoping screws and its lateral protrusion.

**Results:**

Pre-operative mean FIM™ and NMS were 4.3 (range 1–9) and 98.7 (range 22–126), respectively. At the 12-month follow-up the average FIM™ and NMS were 95.3 (range 22–126) and 3.7 (range 1–9), respectively. Mean shortening of the lag screws was 4.3 mm (range 1–8) and mean lateral protrusion was 1.7 mm (range 0–3). 3 Cases (0.70%) of non-consolidation requiring reoperation were recorded. 1 Case (0.24%) of these cases was also characterized by nail breakage. No case of cut-out has been reported at our follow-up.

**Conclusions:**

This dual telescoping nail system is effective and safe. The sliding of the telescoping screws within the barrel is able to decrease strain from the femoral head during weight bearing reducing the risk of cut-out.

## Introduction

Hip fractures in the elderly have a significant socio-economic impact due to their frequency, disability, and mortality [1–3]. The preferred treatment for trochanteric fractures has evolved over time resulting in the cephalomedullary nail (CMN) to be the current gold standard because of its reported biomechanical advantages compared to sliding hip screws [4–6]. However, failures and complications related to the lag screw mechanism are still frequent and may include cut-out, back out, medial migration, excessive lateral migration, femoral neck shortening, and *Z*-effect when two screws are used [7–10]. Recently, a new telescoping dual lag screw nail system (Chimaera trochanteric nail—Orthofix®, Lewisville, Texas, USA) was designed to allow the lag screws to slide within a fixed barrel when compressed and to lock the lag screws to the nail without a set of screw.

This feature is intended to improve rotational stability and minimize the risk of screw cut-out, by providing immediate linear compression and allowing safe weight bearing during the process of fracture consolidation of the osteoporotic bone.

Currently, there is a paucity of standardized biomechanical evidence in the existing literature regarding the impact of this biomechanical solution on complication rates.

A meta-analysis comparing clinical outcomes of InterTan and Gamma nail or PFNA in the treatment of intertrochanteric fractures reported significant differences in implant cut-out, favoring the dual lag screw system [11].

Therefore, the aim of this study was to evaluate whether a dual telescoping lag screw nailing system is associated with a reduced rate of failure and complications in patients treated for proximal femoral fractures with intramedullary nailing.

## Materials and methods

### Study design and participants

This study presents a single cohort retrospective analysis of prospectively reported data of 742 consecutive patients undergoing closed reduction and internal fixation for intertrochanteric fracture at a single Institution between January 2017 and June 2020. This study was conducted in accordance with the STROBE (Strengthening the Reporting of Observational Studies in Epidemiology) guideline for cohort studies. The study design, data collection, and reporting followed the recommended items outlined in the STROBE checklist to ensure transparency and rigor in the conduct of observational research. Inclusion criteria were: patients with AO-OTA proximal femoral fractures 31A.A1-A3 treated using the short (180 mm) Chimaera trochanteric nail (Orthofix® SRL, Lewisville, Texas, USA) with telescopic lag screw and supplementary telescopic screw configuration, and a follow-up of at least 12 months were included. Participants who did not provide consent or expressed unwillingness to participate were excluded.

To address potential confounding factors and effect modifiers, the following exclusion criteria were implemented: individuals aged < 65 years old, previous ipsilateral hip or femur surgery, patients unable to walk before fracture and patients with medical contraindications, medical illness or cognitive disorders precluding participation to follow-up examinations (such as dementia, Parkinson disease, Alzheimer disease, severe heart failure, kidney disease requiring dialysis, neoplasm).

After screening 742 in our database against the inclusion and exclusion criteria, a total of 567 patients were deemed eligible for inclusion in the study.

The institution Internal Review Board (IRB) approved the human protocol for this investigation (registration number NCT06285981); all investigations were conducted in conformity with ethical standards of the institutional and national research committee and with the 1964 Helsinki Declaration and its later amendments. All patients have given their informed consent for participation.

### Clinical pre-operative and post-operative assessment

Pre-operative and post-operative functional evaluation was assessed through the Functional Independence Measure (FIM™) instrument and the Parker’s New Mobility Score (NMS) [12, 13]. The FIM™ instrument is a basic indicator of the severity of the disability composed by 18 items divided into two major groups (the Motor items and the Cognitive Items) with a total score ranging from 18 to 126. The NMS assesses the functional level of the patient by a total score ranging between 0 (no walking ability at all) to 9 (fully independent).

All patients received the same rehabilitation protocol with early mobilization. Full weight-bearing on the affected side was allowed starting from post-operative day-one, unless intra-operative complications occurred that restrained or delayed weight-bearing. Full weight-bearing was also delayed when patients were not able to stand up on the first day postoperatively due to factors such as clinical condition, compliance, or the ability to actively engage in the rehabilitation protocol from post-operative day 1. Nevertheless, every patient received consistent encouragement and motivation to stand up as an integral aspect of the rehabilitation process.

Post-operative functional evaluation included the patients’ walking speed assessment using the Timed Up and Go (TUG) test and the evaluation of the FIM™ and the NMS during the follow-up [14].

### Radiographic pre-operative and post-operative assessment

Pre-operative, intra-operative and post-operative radiographic evaluation included standard antero-posterior (AP) and latero-lateral (LL) projections. Intra-operative radiological examination was performed for each patient at the end of the surgical operation. All the radiological measurements were performed through TraumaCad (Brianlab, Munich, German) software.

**Fig. 1:**
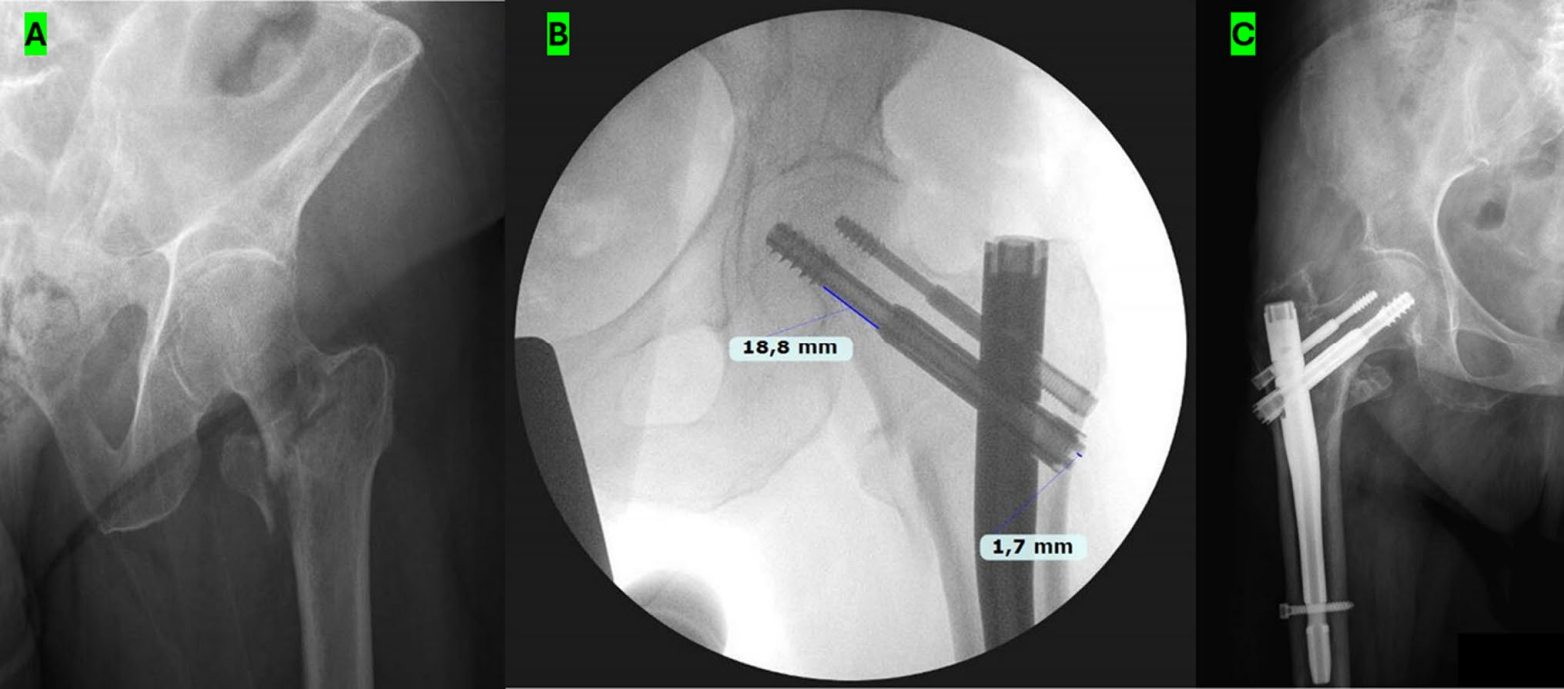
Chimaera trochanteric nail (Orthofix®, Lewisville, Texas, USA) comes in a 180 mm length and it is suitable for both, left and right femur. The proximal locking holes allow the placement of a lag screw and a second supplementary screw to provide additional rotational stability. The lag screw connects to the nail by a specifically designed crown that is part of the screw. **A** AO-OTA 31-A2.2 pertrochanteric fracture with detachment of the lesser trochanter. **B** The TAD distance is calculated by the sum of the distances from the tip of the lag screw to the apex of the femoral head on AP and LL radiograph after adjusting for magnification. Goal TAD is < 25 mm. **C** Plain AP radiograph showing the femoral bone healed after a pertrochanteric fracture treated with CRIF. Bone healing process after intramedullary nail fixation occurs by endochondral ossification. The shortening of the lag screws and the lateral protrusion of the sliding screws calculated on the AP projection. This feature of the nail is intended to minimize the risk of screw cut-out, by providing immediate linear compression and allowing safe weight bearing during the process of fracture consolidation

All fractures were classified according to the AO-OTA classification (Fig. 1A). Radiological follow-up included the degree of the reduction according to the Baumgartner criteria, the tip-apex distance, and the shortening of the telescoping screws and its lateral protrusion (Fig. 1B) [15, 16].

According to Baumgartner’s criteria, the reduction was considered “good” when a displacement of any fragment inferior to 4 mm, a normal or mild valgus alignment on the AP view, and an angle of less than 20° between the axis of the femur and the neck/head on the LL view were achieved. The reduction was considered “sufficient” when alignment or displacement criteria for a “good” reduction were met, but not for both. “Poor” reduction was considered when none of the criteria for angulation or displacement were obtained.

Complete bone healing was evaluated considering the alignment of the fragments, the presence of mature bone callus, and the disappearance of the fracture line (Fig. 1C).

The measure of the TAD was performed by calibrating the X-ray on the known lag screw diameter. The value of the TAD was then given by the sum of the distance from the apex of the lag screw to the apex of the femoral head in the AP and LL projection.

By calibrating the screw diameter, the correct extent of shortening and lateral protrusion of the main lag screw on the AP view were measured. The shortening of the lag screws was measured as the sliding distance between the start of the thread and the static portion of the screw, and the lateral protrusion of the sliding screws was calculated on the AP projection as the distance, along the superior border of the screw, between the lateral femoral cortex and the lateral end of the screw (Fig. 1B). Lateral protrusion and screw shortening obtained from the immediate post-operative X-ray were then compared with those of the last follow-up.

Any complication was recorded at the final outpatient assessment.

### Statistical analysis

Descriptive statistics, such as means and ranges, were calculated for continuous variables; while, categorical variables were summarized using frequencies and percentages. Missing data were assessed and found to be missing completely at random. Given this assumption, a pairwise deletion approach was employed, where missing values were excluded on a pairwise basis during the analyses. This decision was made to utilize all available data for each specific analysis while minimizing potential biases associated with missing data. The Kolmogorov–Smirnov test was used to assess normality of the data. Paired *t*-test was used for comparison of continuous variables for normal distributed variables. The Wilcoxon signed-rank test was used for comparison of continuous variables for non-normal distributed variables. Statistical significance was set at *p* < 0.05.

Statistical analysis and data collection was performed using SPSS v22 (IBM SPSS Statistics, Armonk, New York, USA).

## Results

**Fig. 2:**
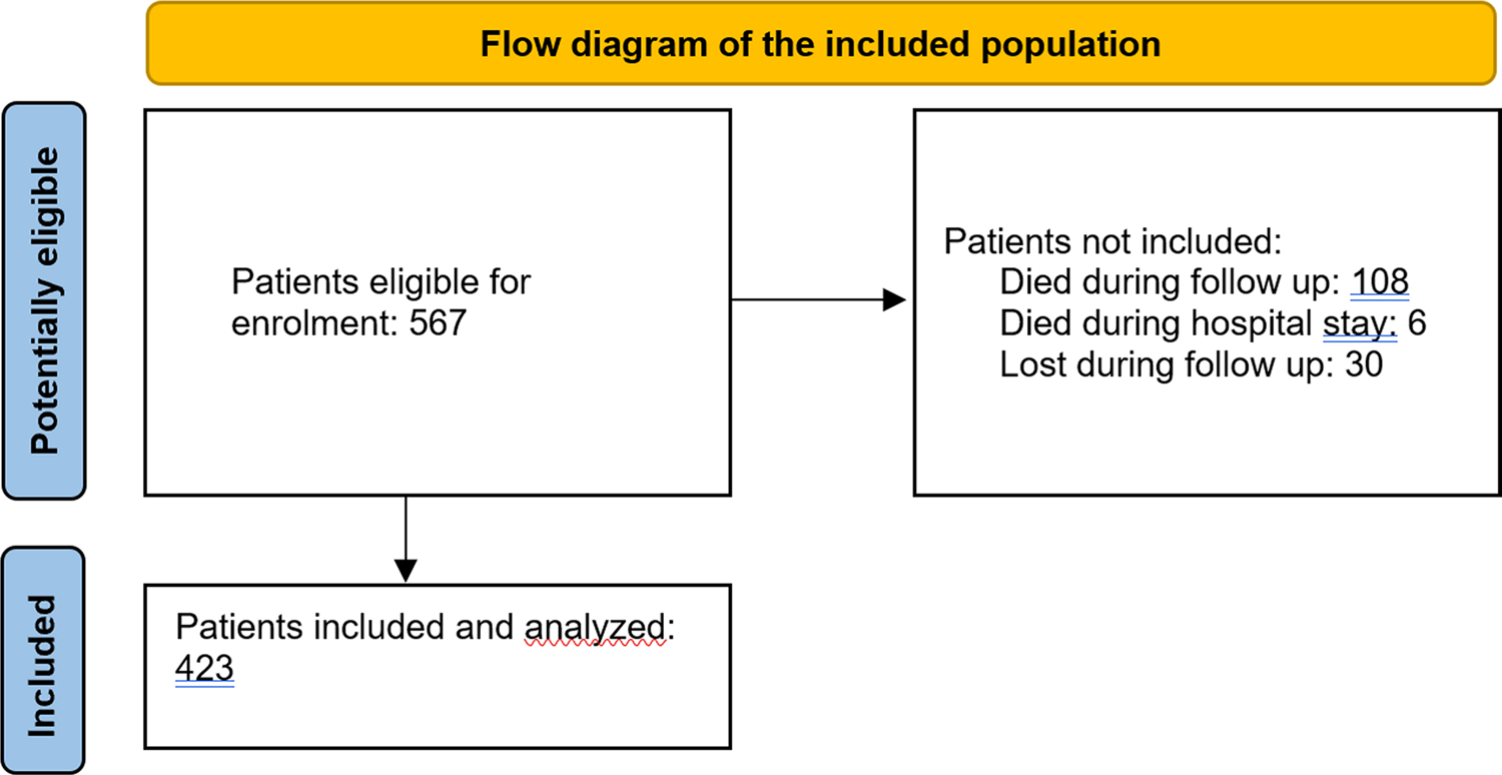
Flow diagram of the included population according to STROBE guidelines

742 patients with AO-OTA 31-A intertrochanteric fractures were enrolled at a single Institution. After screening 742 in our database against the inclusion and exclusion criteria, a total of 567 patients were deemed eligible for inclusion in the study. Of the 567 patients enrolled and eligible for inclusion, 6 died in hospital (1.06%) and 108 died during follow-up (19.05%) and 30 (5.29%) were lost during follow-up within a year. Overall, a final group of 423 patients were included and analyzed with a minimum follow-up of 12 months (mean 18.2 months; range 12–26 months) as shown in the flow diagram in Fig. 2. The mean age was 83.9 ± 12.88 (range 65–95); 72 were male (17.02%) and 351 (82.97%) were female; the affected side was the right in 218 patients (51.53%) and the left in 205 patients (48.46%); the mean Body Mass Index (BMI) was 23.2 ± 3.96 (range 18–31). Demographic data are reported in Table 1.

**Table 1:** Demographic data of the study population

Demographic variables		
Age (y.o)	mean ± SD	83.9 ± 12.88
	[min, max]	[65–95]
BMI (kg)	mean ± SD	23.2 ± 3.91
	[min, max]	[18–31]
Gender, *n* (%)	72 males (17.0%)	351 females (83%)
Affected side, *n* (%)	218 right (51.5%)	205 left (49.5%)
Cardiovascular events	*n* (*%*)	86 (20.3)
Smokers		40 (9.4)
Type 2 Diabetes Mellitus	*n* (*%*)	78 (18.4)
Hypertension	*n* (*%*)	250 (59)
Hypothyroidism	*n* (*%*)	56 (13.2)
Anemia	*n* (*%*)	41 (9.6)
Atrial Fibrillation	*n* (*%*)	70 (16.5)
Chronic Kidney Disease	*n* (*%*)	31 (7.3)
Dialysis	*n* (*%*)	4 (0.9)
Heart Failure	*n* (*%*)	41(9.6)
COPD	*n* (*%*)	37 (8.7)
Lipids	*n* (*%*)	54 (12.7)

*BMI* body mass index

**Table 2:** Pre- and post-operative student *t*-test for FNM and MIS

Outcome	Pre-operative	Postoperative	*t* test	*P* value
FNM	98.7	95.3	0.428	0.995
MIS	4.3	3.7	0.232	0.918

According to the AO-OTA classification we reported 245 (57.91%) stable fractures (type A1.1-3), 142 (33.56%) unstable fractures (type A2.2-3), and 36 (8.51%) reverse oblique fractures (type A3.1-3). Fracture type specification is given in Table 2.

A multidisciplinary clinical evaluation, according to our protocol, was performed within 24 h before surgery from admission in 384 patients (90.78%) and resulting in the presence of cardiovascular disease in 286 patients (67.61%), of diabetes mellitus in 117 patients (27.66%), of previous stroke in 72 patients (17.02%), and of Parkinson’s disease in 43 patients (10.16%). Two major co-morbidities were present in 153 patients (36.17%), among which the most frequent were coronary heart disease (72 patients, 17.02%) and heart failure (61 patients, 14.36%).

The average time from admission to intervention was 32 h (range 4123) and 88.29% of patients (373 patients) underwent surgery within two calendar days from admission. Mean operative time, meaning from skin incision to wound closure, was 46 min (range 23–100 min). No intra-operative complications were reported.

### Clinical outcomes

During hospital stay, a total of 205 clinical complications were recorded, including 85 blood transfusions, 40 deep vein thrombosis, 4 wound infection, 14 pneumonia, and 61 urinary tract infections.

In-hospital mortality was 1.06% (6/567); while, 12-month mortality was 25.5% (108/423).

**Fig. 3:**
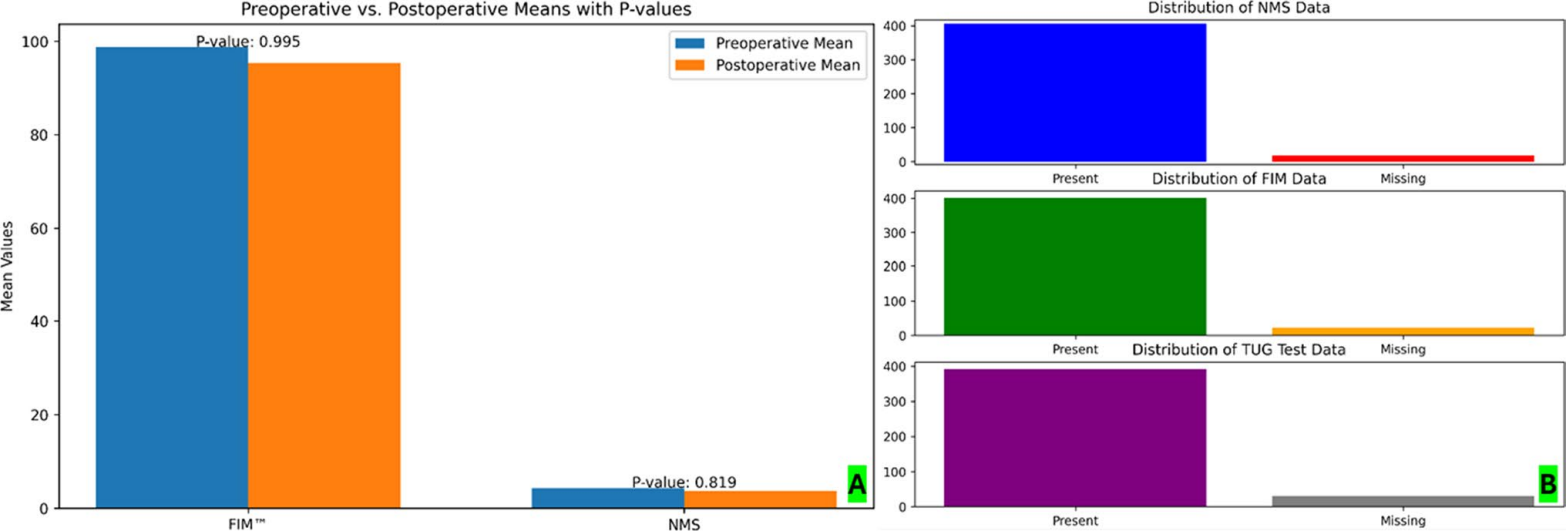
**A** Pre- and post-operative student *t*-test for FNM and MIS shows no statistical differences. **B** Bar chart of missing values for clinical outcomes

Pre-operative mean FIM™ and NMS were 4.3 (range 1–9) and 98.7 (range 22–126), respectively. At the 12-month follow-up the average FIM™ and NMS were 95.3 (range 22–126) and 3.7 (range 1–9), respectively. Student *t*-test showed no statistically significant differences between pre and post-operative results for FIM™ and NMS as shown in Table 3 (Fig. 3A). 320 patients (75.6%) passed the TUG test at the last follow-up and the average value of the TUG test was 74 s. Among the outcomes assessed, missing values were noted for NMS, FIM, and TUG test, with 17, 23, and 31 instances, respectively, in a cohort of 423 individuals as shown in Fig. 3B. Notably, no missing data were reported for other radiological outcomes in the study.

### Radiographic outcomes

According to Baumgartner’s criteria, a “good” reduction was obtained in 146 patients (34.5%), “sufficient” in 178 patients (42.0%), and “poor” in 99 patients (23.4%). Advanced bone healing was observed in 37.82% of patients (160) at 6 weeks follow-up; complete bone healing was observed in 99.29% of fractures (420) at 6 months follow-up.

The average TAD was 22.27 mm (range 9.8–42 mm; SD 6.49) intraoperatively and 21.96 mm (range 10.5–41.2 mm; SD 6.18) at the last follow-up (*P* = *0.34)*; 71.4% of cases showed a TAD < 25 mm.

**Fig. 4:**
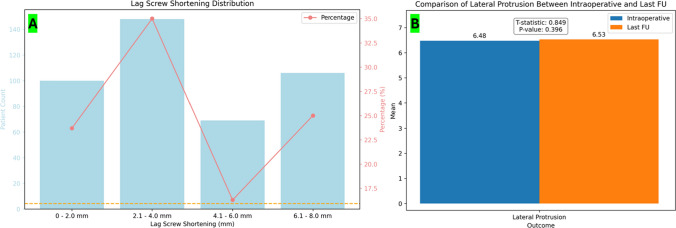
**A** Pre- and post-operative student *t*-test for lag screw shortening at the last follow-up. The mean lag screw shortening at the last follow-up was 4.35 mm (range 1–8 mm; SD 2.18), with a value between 2.1 mm and 4 mm in 148 cases (34.9%). **B** Pre- and post-operative student *t*-test for lag screw lateral protrusion at the last follow-up. The average lateral protrusion was 6.47 mm (range 1–15 mm; SD 2.81) intraoperatively and 6.53 mm (range 1–16; SD 2.79) at the last follow-up with no statistically significant differences (*P* = 0.42). Mean difference between the intra-operative and the last follow-up lateral protrusion was 0.06 mm. A lateral protrusion between 4.1 mm and 8 mm was found in 238 cases (56.26%) intraoperatively and in 244 cases (57.68%) at the last follow-up. 18 patients (4.2%) reported clinical lateral hip pain

Mean shortening of the lag screws was 4.3 mm (range 1–8) and mean lateral protrusion was 1.7 mm (range 0–3) (Fig. 4). 1 case (0.24%) of these cases was also characterized by nail breakage. No case of cut-out has been reported at our follow-up. Fracture consolidation in remaining patients occurred within 36 weeks (range 4–36).

**Table 3:** Distribution of proximal femur fractures according to AO-OTA Classification

Proximal femur AO-OTA classification (2018)	0.1	0.2	0.3	Tot. *n* = *423*
31A1—stable, *n* (%)	0 (0%)	69 (16.3%)	175 (41.3%)	244 (57.6%)
31A2—unstable, *n* (%)	No more contemplated	94 (22.2%)	48 (11.3%)	142 (33.5%)
31A3—reverse oblique, *n* (%)	30 (7.1%)	0 (0%)	6 (1.4%)	36 (8.5%)

## Discussion

In this retrospective analysis of prospectively recorded data involving patients with AO OTA 31.A proximal femoral fractures treated with a dual telescoping nail system, the study reveals favorable clinical and radiological outcomes. Notably, no cut-out was recorded in the study cohort. Another notable finding of the study is the effectiveness of this nail system in limiting lateral protrusion of the lag screw. This observed evidence has implications for clinical outcomes and revision rates, suggesting a potential positive impact on the stability and performance of this type of system.

The most frequent complication of CMN is the cut-out, which is defined as a varus collapse of the neck-shaft angle with consequent protrusion of the lag screw from the femoral head [17].

Risk factors associated with cut-out include a TAD more than 20 mm, a non-anatomical reduction with a varus neck-shaft angle, and a position of the screw in the femoral head that is not in a center-center or center-inferior position according to Cleveland zones [18, 19]. However, cut-out often occurs even with a well-positioned lag screw since this complication is manly related to the biomechanical behavior of the lag screw and the head-neck complex during the healing process.

Controlled sliding of CMN lag screw is crucial to guide the collapse of the fracture fragments until proximal bone rests on a stable and intact distal bone. Sliding enables transmission of forces through the fracture site and enhances fracture consolidation.

However, single lag screw are designed to slide only on the axis of the screw in a superior-medial to an inferior-lateral direction. Unfortunately, it has been widely demonstrated that the movements of the fragments occur in several directions with femoral heads rotating along the femoral neck axis and shifting posteriorly [20]. These movements are a direct consequence to the large and dynamic three-dimensional hip forces and moments of forces affecting the proximal femur even in ordinary day-walking.

For these reasons it is widely accepted that the nature of the cut-out is multidirectional with significant rotational and translational displacements. Therefore, preventing the femoral head to rotate can reduce the possibility of cut-out as a fixed single screw-type represents a disadvantage because it virtually had no ability to do so.

This is not the first telescoping lag screw system and clinical results of telescoping devices have already been published in the literature. The results of the Chimaera trochanteric nail (Orthofix®, Lewisville, Texas, USA) were recently reported by Traverso et al. [21]. The study considered a small cohort of 99 proximal femur fractures treated with the same nail system and reported 1 case of cut-out. However, the study did not cluster the results of the fractures treated with the cephalocervical screws alone and those treated with the supplementary screw. The only patient affected by nail cut-out was treated placing only the cephalocervical lag screw. In the current study the surgical technique was standardized including cases where both the cephalic and the supplementary screws were placed. According to this evidence, the current study could be more accurate assessing the effectiveness of this nail design.

Kawatani et al. [22] reported the radiological outcome of 310 patients treated with a telescoping lag screw combined with an antirotation pin (Targon® PF B.Braun, Aesculap). All fractures healed except for 1 failure occurring from a medial perforation, but no cut-out was recorded.

Parker MJ et al. [23] revealed 3 failures with 2 cut-outs in 215 patients at 1-year follow-up.

Biber et al. [24] reported 1516 patients treated with a proximal femoral Targon® PF nail. Cut-out was observed in 1.1% (CI: 0.5–1.6%) and medial perforation was reported in 0.5% (CI: 0.2–0.9%) of patients.

Jagow et al. [18] in a small series of 41 patients treated with the Galileo Lag Screw System (Advanced Orthopedic Solutions, Torrance, CA) reported 1 femoral head cut-out, which occurred after the lag screw had telescoped its entire distance and began functioning as a rigid non-compressible lag screw.

The telescoping Pertrochanteric Nail (PTN) (Biomet® Ltd, Bridgend, United Kingdom) has been evaluated in cadaver-based studies only [25].

A low incidence of cut-out was shared by the previous studies.

In this series of 423 patients with a minimum of 1 year follow-up, no cut-out was recorded although “sufficient” and “poor” reduction was obtained in 42% and 23.4% of cases, respectively. The TAD could be considered a significant factor for cut-out even in dual lag screw nails and no cases of cut-out are reported in this series, despite a TAD < 25 mm in 71.4% of patients at the last follow-up.

In the current study, mean averaged shortening of the screw was 4.35 mm following the telescoping system without resulting in a lateral screw protrusion due to the self-locking mechanism of the lag screw. 6% of the patients had trochanteric lateral pain, which is far less then reported in the current literature to date.

### Limitations

This study had some limitations. The number of the enrolled patients, despite being the highest presented in the current literature, may be low to highlight rare complications associated with this nail system. Moreover, a mean follow-up of 18.2 months may be too short to detect possible late complications such as avascular necrosis or nail breakage. Another limitations of this study include the involvement of multiple surgeons**.** Another limitation is the pairwise deletion of missing data, which may introduce bias in term of selection bias, impact on statistical power, and increasing type II errors.

### Future directions

Given these promising findings, the author suggests that further investigation through a higher-level evidence study comparing the incidence of cut-out with dual telescoping lag screw nail versus single lag screw may be of significant interest for a more comprehensive understanding of the treatment efficacy.

## Conclusion

This dual telescoping nail system is effective and safe. The sliding of the telescoping screws within the barrel is able to decrease strain from the femoral head during weight bearing reducing the risk of cut-out.
